# A Protocol for Advanced Psychometric Assessment of Surveys

**DOI:** 10.1155/2013/156782

**Published:** 2013-01-15

**Authors:** Janet E. Squires, Leslie Hayduk, Alison M. Hutchinson, Lisa A. Cranley, Mark Gierl, Greta G. Cummings, Peter G. Norton, Carole A. Estabrooks

**Affiliations:** ^1^Clinical Epidemiology Program, Ottawa Hospital Research Institute, Ottawa, ON, Canada K1H 8L6; ^2^School of Nursing, University of Ottawa, Ottawa, ON, Canada K1H 8M5; ^3^Department of Sociology, University of Alberta, Edmonton, AB, Canada T6G 2H4; ^4^Cabrini-Deakin Centre for Nursing Research, School of Nursing and Midwifery, Deakin University and Cabrini Health, Melbourne, VIC 3KM, Australia; ^5^Faculty of Nursing, University of Alberta, Edmonton, AB, Canada T6G 1C9; ^6^Department of Educational Psychology, University of Alberta, Edmonton, AB, Canada T6G 2G5; ^7^Department of Family Medicine, University of Calgary, Calgary, AB, Canada T2M 0H5

## Abstract

*Background and Purpose*. In this paper, we present a protocol for advanced psychometric assessments of surveys based on the Standards for Educational and Psychological Testing. We use the Alberta Context Tool (ACT) as an exemplar survey to which this protocol can be applied. *Methods*. Data mapping, acceptability, reliability, and validity are addressed. Acceptability is assessed with missing data frequencies and the time required to complete the survey. Reliability is assessed with internal consistency coefficients and information functions. A unitary approach to validity consisting of accumulating evidence based on instrument content, response processes, internal structure, and relations to other variables is taken. We also address assessing performance of survey data when aggregated to higher levels (e.g., nursing unit). *Discussion*. In this paper we present a protocol for advanced psychometric assessment of survey data using the Alberta Context Tool (ACT) as an exemplar survey; application of the protocol to the ACT survey is underway. Psychometric assessment of any survey is essential to obtaining reliable and valid research findings. This protocol can be adapted for use with any nursing survey.

## 1. The Alberta Context Tool

Organizational context is “…the environment or setting in which people receive healthcare services, or in the context of getting research evidence into practice, the environment or setting in which the proposed change is to be implemented” ([1], page 299). Context is believed to influence the successful implementation of research evidence by nurses in healthcare settings internationally. However, there is little empirical evidence to support this claim. One reason for this is the absence of a robust measure of organizational context in healthcare. The Alberta Context Tool (ACT) was developed in 2006 to address this gap.

Underpinned by the Promoting Action on Research Implementation in Health Services (PARiHS) framework [1, 2] and related literature [3, 4], the ACT was constructed to measure healthcare providers' and care managers' perceptions of modifiable dimensions of organizational context; their responses can then be aggregated to provide nursing unit and/or organizational (e.g., hospital or nursing home or home care office) estimates of context. Three principles informed the development of the ACT: (1) use of the PARiHS framework and related literature to identify a comprehensive set of contextual concepts, (2) brevity—it could be completed in 20 minutes or less, and (3) a focus on modifiable (and therefore researchable) elements of context [5]. The survey now exists in four versions (acute-adult care, pediatrics, long-term care, and home care) and six forms: (1) regulated nursing care providers—registered nurses and licensed practical nurses; (2) unregulated nursing care providers-healthcare aides; (3) allied health providers; (4) physicians; (5) practice specialists (e.g., clinical educators); and (6) unit care managers. It is being used in eight countries (Canada, United States, Sweden, Netherlands, United Kingdom, Republic of Ireland, Australia, and China) and is available in five languages (English, Dutch, Swedish, Chinese, and French). The index version of the survey (English, acute care regulated nurses) contains 56 items representing eight dimensions and 10 concepts: (1) leadership, (2) culture, (3) evaluation, (4) social capital, (5) informal interactions, (6) formal interactions, (7) structural and electronic resources, and (8) organizational slack (representing three subconcepts: staff, space, and time). Definitions of the eight dimensions, and a description of their operationalization, are presented in Table 1. Content validity (i.e., the extent to which the items adequately represent the content domain of the concept) was established by members of the research team responsible for its development and with expertise in the context field. No quantification (e.g., content validity index) of content validity has been performed to date The instrument was originally developed for acute (adult) care and modified for use in pediatrics, nursing homes, and home care. Response processes validity (i.e., how respondents interpret and expand on item content) was completed in all four settings [6–8].

## 2. Traditional Psychometric Assessment of the Alberta Context Tool

To date, two preliminary traditional psychometric assessments of the ACT have been published [5, 9]. The first assessment used scores obtained from pediatric nurse professionals enrolled in a national, multisite study [5]. In that analysis, a principal components analysis (PCA) indicating a 13-factor solution was reported. Bivariate associations between instrumental research utilization (which the ACT was developed to predict) and a majority of ACT factors as defined by the PCA were statistically significant at the 5% level. Each ACT factor also showed a trend of increasing mean scores ranging from the lowest to the highest level of instrumental research use, adding additional validity evidence. Adequate internal consistency reliability using Cronbach's alpha coefficients was reported; alpha coefficients ranged from 0.54 to 0.91 [5]. A subsequent validity assessment was conducted on responses obtained from healthcare aides (i.e., unregulated nursing care providers) in residential long-term care settings (i.e., nursing homes) [9]. The overall pattern of the ACT data (which was assessed using confirmatory factor analyses) was consistent with the hypothesized structure of the ACT. Additionally, eight of the ten ACT concepts were related, at statistically significant levels, to instrumental research utilization, supporting its validity. Adequate internal consistency reliability was again reported with alpha coefficients for eight of ten concepts exceeding the accepted standard of 0.70 [9]. Additional details on both of these preliminary assessments is available elsewhere [5, 9].

There are now sufficient ACT data collected from nursing care providers (i.e., registered nurses, licensed practical nurses, and healthcare aides) and allied healthcare professionals across a variety of healthcare settings to conduct advanced psychometric assessments on scores obtained with the instrument. This will allow researchers and decision makers to use the survey, with greater confidence, to inform the design and evaluation of context-focused interventions as a means of improving research use by nursing care and allied providers. In this paper, we present a protocol for advanced psychometric assessments of surveys that is based on the Standards for Educational and Psychological Testing (i.e., the Standards). We use the ACT, for which this protocol was developed, as an exemplar survey of which this protocol can be applied. Application of the protocol to the ACT is currently underway.

## 3. A Protocol for Advanced Psychometric Assessment

The Standards, considered best practice in the field of psychometrics [10], follows closely the work of American psychologist Samuel Messick [11–13], who viewed validity as a unitary concept with all validity evidence contributing to construct validity. Validation, in this framework, involves accumulating evidence from four sources (content, response processes, internal structure, and relations to other variables) to provide a strong scientific basis for proposed score interpretations. It is these interpretations of scores that are then evaluated for validity, not the instrument itself. The source(s) of evidence sought for any particular validation is determined by the desired interpretation(s) [14]. Content evidence refers to the extent to which the items included in an instrument adequately represent the content domain of the concept of interest. Response processes evidence refers to empirical evidence of the fit between the concept under study and the responses given by respondents on the item(s) developed to measure the concept. Internal structure evidence examines the relationships between an item set. Relations to other variables evidence examines relationships between the concept of interest (e.g., the 10 concepts in the ACT) and external variables (e.g., research utilization in the case of the ACT) that it is expected to predict or not predict, as well as relationships to other scales hypothesized to measure the same concept(s) [15].

Our psychometric protocol specifically addresses: (1) data preparation (which is often necessary to reconfigure and merge multiple datasets to conduct advanced and rigorous psychometric analyses; there is little guidance in the literature on how to do this) and (2) advanced psychometric data analyses that are in line with the Standards. Robust psychometric analysis of survey data should involve examining the data for: (1) validity, (2) reliability, and (3) acceptability [16–18]. Therefore, this protocol includes each of these components. Validity refers to the extent to which a measure achieves the purpose for which it is intended, and is determined by the “degree to which evidence and theory support the interpretations of test scores entailed by proposed users of tests” ([15], page 9). Reliability refers to the consistency of measurement obtained when using an instrument repeatedly on a population of individuals or groups [15]. Acceptability refers to ease of use of an instrument [17]. While multiple reports and descriptions of these analyses can be located in the literature [15–17], several limitations are noted. First, there has been no attempt to synthesize the information into a usable protocol. Second, few reports mention acceptability, which is a core component of psychometrics. Third, most current psychometric literature in nursing and health services research includes descriptions of analyses based solely on Classical Test Score Measurement Theory and that are “exploratory” in nature. For example, few reports explore alternatives to traditional (Cronbach's alpha) reliability testing. A rigorous assessment of reliability should go beyond Cronbach's alpha and also include an assessment of variances or standard deviations of measurement errors and item and test/scale information functions (using Item Response or Modern Measurement Theory). Finally, with respect to validity, most publications limit their discussion to “types” of validity and report methods of limited robustness such as correlations and principal components analysis; little attention is given to rigorous multivariate assessments such as regression and structural equation modeling.

A central reason we chose the Standards as the guiding framework for our protocol is because it provides a contemporary view of validity. Traditionally, three types of validity are often discussed: content validity, criterion-related validity (which included concurrent and predictive validity), and construct validity. This holy trinity conceptualization of validity as labeled by Guion [19] has dominated nursing and health-related research method textbooks. While this way of conceptualizing validity has been useful, it has also caused problems and confusion. For example, it has led to compartmentalized thinking about validity, narrowing and limiting it to a checklist type of approach. It has made it “easier” to overlook the fact that construct validity is really the whole of validity theory, that is, that validity is really a unitary concept. It has also resulted in validity being viewed as a property of the measure (instrument) rather than a property of the scores obtained from a measure when it is used for a specific purpose with a particular group of respondents. Therefore, in the psychometric protocol (presented next), we take a unitary approach to validity assessment.

## 4. Methods

The psychometric protocol presented in this paper addresses all three core components of survey psychometrics: acceptability, reliability, and validity. We focus on advanced aspects of validity (i.e., internal structure and relations with other variables' validity evidence) in order to construct robust validity arguments for survey data. The protocol is divided into two phases: (1) data preparation and (2) data analysis. These phases will be applicable to psychometric assessment of all multi-item survey instruments.

### 4.1. Phase I: Data Preparation

Robust psychometric assessment often requires the combination of multiple data collections. We will conduct a psychometric analysis of ACT data across seven unique data collections (See Table 2). The data comprise: (1) various provider groups (healthcare aides, licensed practical nurses, registered nurses, and allied healthcare professionals); (2) settings: (adult hospitals, pediatric hospitals, nursing homes, and community care); and (3) survey administration modes (pen and paper, online, and computer assisted personal interview). In addition to data on the ACT, some of these collections also contain data on knowledge translation (defined as research utilization, which the ACT was developed to predict), individual factors (e.g., attitude towards research), care provider outcomes (e.g., burnout), and patient/resident outcomes (e.g., number of falls) which context (through research utilization) is hypothesized to predict. These additional variables are necessary to perform advanced psychometric analyses on the ACT. Demographic data files accompany all seven data collections. Collections 1–6 include items on knowledge translation; collections 1–4 include items on care provider outcomes; and collections 1–4 include data on patient/resident outcomes.

The first phase of completing a comprehensive psychometric assessment using survey data from multiple sources is “data preparation”. Substantive work is often required to reconfigure multiple data collections for psychometric analysis. In the case of the ACT, we needed to merge data by provider subgroup to allow for separate (homogenous) analyses for healthcare aides, nurses, and allied healthcare professionals. This work involves detailed “mapping” of survey elements of all data files to link items (including lead-ins, stems, and examples of concepts where they exist) and response scales across each data file by provider subgroup, setting, and survey administration mode. The research team needs to meet regularly to discuss the mapping and address any concerns regarding where items can and cannot be combined to facilitate merging of data files to create a file from which the psychometric analyses can be conducted. With the ACT, survey elements mapped included: interviewer instructions (where a computer assisted interview was undertaken in data collection), lead-in statements (e.g., In answering the following, please focus on….), stems (the standard introduction to the items), examples (e.g., number of resident falls is an example of the context concept of evaluation), survey items, response options, skip pattern instructions, and the order of items within an item set for a concept.

### 4.2. Phase II: Data Analysis

All initial analyses described next will, in the case of ACT, be conducted for each provider subgroup: regulated nursing care providers (registered nurses, licensed practical nurses), unregulated nursing care providers (healthcare aides), and allied healthcare professionals. Subsequent analyses will be informed by initial analyses and may vary by provider group. Our aims with respect to psychometric assessment of the ACT (and those which frame our protocol) are as follows.

1. To assess advanced psychometric properties of the ACT for regulated and unregulated nursing care providers and allied health providers by:
  - setting (adult and pediatric hospitals, nursing homes, home care), and
  - mode of administration (pen and paper, online, computer assisted personal interview);
2. To test the theoretical model underpinning the ACT; and
3. To assess performance of the ACT when data are aggregated to higher (e.g., nursing unit and organizational/hospital) levels.

These aims are applicable to psychometric assessment of most survey instruments.

### 4.3. Objective 1: To Assess the Psychometric Properties of the ACT by Provider Subgroup, Setting, and Mode of Administration

#### 4.3.1. Acceptability

We will assess acceptability of the ACT by examining missing data frequencies for all items and subscales (concepts). We will also assess, where available, the time taken to complete each subscale and the full survey [17, 18, 20].

#### 4.3.2. Reliability

Reliability information may be reported in terms of variances or standard deviations of measurement errors, in terms of item response theory test/item information functions, or more commonly, in terms of one or more coefficients. We will assess reliability by calculating internal consistency and information functions. We will calculate three internal consistency coefficients: (1) Cronbach's alpha; (2) Guttman split-half reliability; and (3) Spearman-Brown reliability. Internal consistency coefficients are indexes of reliability associated with the variation accounted for by the true score of an “underlying concept” [17], in our case, each ACT concept. Coefficients can range from 0 to 1; a coefficient of 0.70 is considered acceptable for newly developed scales while 0.80 or higher is preferred and indicates the items may be used interchangeably [17, 20]. Information functions are a function of discrimination and item thresholds in item response theory; they present the amount of information provided by an item at a given trait level [21].

#### 4.3.3. Internal Structure Validity

We will conduct (1) item to total correlations on each ACT concept, (2) item total statistics on each ACT concept (see Table 1 for number of items in each ACT concept), and (3) confirmatory factor analyses (CFA) on each ACT concept and on all ACT items combined.

From the item to total correlations, items will be flagged for discussion and further evaluation if an item correlates with its scale (concept) score below 0.30 [20]. From item-total statistics, items that, if removed, cause a substantial change in the scale Cronbach's alpha score will also be evaluated further and considered for future revision [22].

In developing the ACT, items were chosen to reflect coordinated and meaningfully similar dimensions, but were intentionally chosen to be non-redundant. Hence, the ACT does not exactly match the unidimensional causal requirement of the factor model (tested by CFA). However, the coordination or clustering of meaningfully similar items by substantive similarity, and relevance to potential interventions, render factor specifications the closest statistical model for testing the ACT's internal structure. Further, the similarity of items within each contextual dimension (e.g., leadership, culture, evaluation) renders the CFA approach appropriate for a Standards assessment. We will therefore use CFA to determine how well the defined measurement models for each ACT concept (and all ACT items combined) fit our observed data. A 4-step approach will be used as follows.

1. Model specification (the proposed measurement model for each ACT concept and the complete ACT will be specified),
2. parameter estimation (maximum likelihood estimation will be used),
3. assessment of model fit, and
4. model modification and retesting (as appropriate).

With respect to model fit, we will evaluate parameter estimates for direction, magnitude and significance of effects. Recent discussions of structural equation model testing [23, 24], state chi-square is the only appropriate model test, and have questioned the justifiability of fit indices such as the root mean square error of approximation (RMSEA), the standardized root mean squared residual (SRMSR), and the comparative fit index (CFI). While we are inclined to agree with the critiques of the indices, we are hesitant to entirely disregard them due to their previous popularity and use [18, 25, 26]. Given the shifting statistical view of indices, we will report relevant index values in addition to chi-square to assist comparison to published measurement assessments but we will be cautious about basing conclusions on fit indices.

#### 4.3.4. Relations with Other Variables Validity

Prior to using modeling techniques to test the theoretical model underpinning the ACT (Objective 2), we will examine each ACT item (by scale) for its association with our demographic and dependent variables in the respective datasets (e.g., with research utilization and outcome variables such as healthcare provider health status and burnout). The statistical measure used will depend on the measurement level of the other variable (e.g., a correlation coefficient will be used to examine associations between ACT items and research use). Items within the same scale should correlate at similar magnitudes with the other variables being assessed. Items within a scale that display a pattern uncharacteristic of the other items in the same scale will be further scrutinized with respect to their relations with additional variables.

### 4.4. Objective 2: To Test the Theoretical Model Underpinning the ACT

The ACT was developed based on the premise that a more favorable context leads to higher research use and improved health outcomes of healthcare providers and consequently, improved patient and resident health outcomes (through research use). We will empirically test this theoretical premise using regression and structural equation models. We will construct a series of regression models that examine the relationships between the dimensions of the ACT as independent variables, and research utilization and other outcomes (e.g., care provider burnout) as dependent variables. We will then test a series of structural equation models (SEM) to empirically validate the theoretical (latent-level) model underpinning the ACT. This will allow us to advance our psychometric assessment by simultaneously assessing *both* the measurement and the latent structures of the ACT.

Our SEM models will be specified for each provider subgroup and tested according to the various: (a) settings (adult hospitals, pediatric hospitals, nursing homes, and home care) and (b) survey administration modes (where sample size is sufficient). The models will include demographic variables (as exogenous variables), ACT variables (as endogenous variables), and outcome variables, for example, research utilization (as final endogenous variables). We will follow the same 4-step approach previously identified for CFA:

1. model specification (the proposed measurement model for each ACT concept and the complete ACT will be specified),
2. parameter estimation (maximum likelihood estimation will be used),
3. assessment of model fit, and
4. model modification and retesting (as appropriate).

### 4.5. Objective 3: To Assess the Performance of the ACT with Data Aggregated by Provider Subgroup to Care Unit and Organizational Levels

When developing the ACT, items within the various scales were constructed to direct respondents' attention to common experiences on a particular nursing unit or organization (hospital, nursing home, or residential home/office depending on the context of their care delivery) in order to ensure that the ACT was meaningful at these levels. As a final test of reliability and validity, we will assess performance of the ACT scales when aggregated to the nursing unit and organizational level by calculating four indices: ICC(1), ICC(2), *η* ^2^, and *ω* ^2^. One-way analysis of variance (ANOVA) will be performed on each ACT scale (concept) using the unit as the group variable. The source table from the one-way ANOVA will be used to calculate the four standard aggregation indices [27]. ICC(1) is a measure of individual score variability about the subgroup mean. ICC(2) is an overall estimate of the reliability of group means and provides an index of mean rater reliability of the aggregated data [27]. *η* ^2^, and *ω* ^2^ are measures of validity, also known as measures of “effect size” in ANOVA. An effect size is a measure of the strength of the relationship between two variables and thus, illustrates the magnitude of the relationship. *η* ^2^ denotes the proportion of variance in the individual variable (in each ACT concept) accounted for by group membership (e.g., by belonging to a specific nursing unit) [28]. This value is equivalent to the *R*-squared value obtained from a regression model, and where group sizes are large, to ICC(1) [29]. Omega (*ω*) measures the relative strength of aggregated data as an independent variable. It is also an estimate of the amount of variance in the dependent variable (e.g., in each ACT concept) accounted for by the independent variable (i.e., by group membership-belonging to a specific nursing unit) [30]. Larger values of *η* ^2^ and *ω* ^2^ indicate stronger effect sizes and relationships between variables. As a result, larger values of *η* ^2^ and *ω* ^2^ also indicate stronger “relations to other variables” validity evidence (as described in the Standards validation framework) and thus, contribute to overall construct validity.

## 5. Conclusion

Assessment of the psychometric properties of scores obtained with a survey is critical to obtaining reliable and valid research findings. In this paper, we present a protocol for advanced psychometric assessments of surveys that is based on the Standards for Educational and Psychological Testing (the Standards), considered “best practice” in instrument development and psychometrics [10]. We believe this protocol can be applied to all nursing and related surveys that contain likert-type multi-item scales. Knowing the psychometrics of a survey will, in turn, allow researchers to have greater confidence in their findings and use them to inform the design and evaluation of subsequent phases of their research such as in interventions to improve nursing care and patient outcomes. In this paper, we illustrated the newly developed psychometric protocol using the Alberta Context Tool (ACT) as an exemplar survey to which it can be applied; application of the protocol to the ACT survey is currently underway.

## Figures and Tables

**Table 1:** Dimensions in the ACT survey.

Dimension	Definition	No. Items	Sample Item	Scaling
Leadership	The actions of formal leaders in an organization (unit) to influence change and excellence in practice, items generally reflect emotionally intelligent leadership	6	The leader calmly handles stressful situations	Likert Agreement: (1) strongly disagree (2) disagree (3) neither agree or disagree (4) agree (5) strongly agree

Culture	The way that “we do things” in our organizations and work units; items generally reflect a supportive work culture	6	My organization effectively balances best practice and productivity	Likert Agreement: (1) strongly disagree (2) disagree (3) neither agree or disagree (4) agree (5) strongly agree

Evaluation	The process of using data to assess group/team performance and to achieve outcomes in organizations or units (i.e., evaluation)	6	Our team routinely monitors our performance with respect to the action plans	Likert Agreement: (1) strongly disagree (2) disagree (3) neither agree or disagree (4) agree (5) strongly agree

Social Capital	The stock of active connections among people. These connections are of three types: bonding, bridging, and linking	6	People in the group share information with others in the group	Likert Agreement: (1) strongly disagree (2) disagree (3) neither agree or disagree (4) agree (5) strongly agree

Informal Interactions	Informal exchanges that occur between individuals working within an organization (unit) that can promote the transfer of knowledge	7	Someone who champions research and its use in practice	Frequency Scale: (1) never (2) rarely (3) occasionally (4) frequently (5) almost always

Formal Interactions	Formal exchanges that occur between individuals working within an organization (unit) through scheduled activities that can promote the transfer of knowledge	5	Team meetings	Frequency Scale: (1) never (2) rarely (3) occasionally (4) frequently (5) almost always

Structural/ Electronic Resources	The structural and electronic elements of an organization (unit) that facilitate the ability to assess and use knowledge	11	Notice boards	Frequency Scale: (1) never (2) rarely (3) occasionally (4) frequently (5) almost always (6) not available

Organizational Slack: Human Resources (Staffing)	The cushion of actual or potential resources which allows an organization (unit) to adapt successfully to internal pressures for adjustments or to external pressures for changes	2	Enough staff to deliver quality care	Likert Agreement: (1) strongly disagree (2) disagree (3) neither agree or disagree (4) agree (5) strongly agree

Organizational Slack: Space		4	Time to do something extra for patients	Likert Agreement: (1) strongly disagree (2) disagree (3) neither agree or disagree (4) agree (5) strongly agree

Organizational Slack: Time		3	Use of designated space	Frequency Scale: (1) never (2) rarely (3) occasionally (4) frequently (5) almost always

**Table 2:** Data collections.

Data file no.	Study name funded by	Group(s)	Setting	Country	Mode	Sample size	Data collection
1						HCAs n = 1493 Nurses *n* = 286 Allied *n* = 119 Specialists *n* = 25 Physicians *n* = 9 Managers *n* = 55	Year 1 = 06/2008– 07/2009
2	Translating Research in Elder Care (Project 1) CIHR	HCAs Nurses Allied Specialists Physicians Managers	LTC	Canada	Paper, Online	HCAs *n* = 1506 Nurses *n* = 325 Allied *n* = 156 Specialists *n* = 22 Physicians *n* = 15 Managers *n* = 71	Year 2 = 06/2008– 07/2009

3						Nurses *n* = 764 Allied *n* = 209 Specialists *n* = 51 Physicians *n* = 82 Managers *n* = 32	Year 1 = 05/2008- 06/2008
4	The CIHR Team Grant in Children's Pain (Project 2) CIHR	Nurses Allied Specialists Physicians Managers	Acute Pediatric Hospitals	Canada	Online	Same as above	Year 2 = 05/2011- 06/2011

5	The Role of PDAs in EBP Ministry of Health and Long-Term Care (MOHLTC)	Nurses	LTC Adult Hospitals Pediatric Hospitals Home Care	Canada	Online	*N* = 702	04/2009– 03/2010

6	Linking Best Practice Guideline Use and Health Outcomes for Better Information and Care in the Community MOHLTC Dianne Doran (University of Toronto, Canada)	Nurses	Home Care	Canada	Paper, Online	*N* = 348	04/2009– 03/2010

7	The Older Person and Improving Care (TOPIC 7) (University of South Australia, Adelaide, Australia)	Nurses	Acute Adult Hospitals	Australia	Paper	*N* = 224	09/2008– 12/2008
